# Errors in CGAP xProfiler and cDNA DGED: the importance of library parsing and gene selection algorithms

**DOI:** 10.1186/1471-2105-12-97

**Published:** 2011-04-15

**Authors:** Andrew T Milnthorpe, Mikhail Soloviev

**Affiliations:** 1 School of Biological Sciences, Royal Holloway, University of London, Egham, Surrey, TW20 0EX, UK

## Abstract

**Background:**

The Cancer Genome Anatomy Project (CGAP) xProfiler and cDNA Digital Gene Expression Displayer (DGED) have been made available to the scientific community over a decade ago and since then were used widely to find genes which are differentially expressed between cancer and normal tissues. The tissue types are usually chosen according to the ontology hierarchy developed by NCBI. The xProfiler uses an internally available flat file database to determine the presence or absence of genes in the chosen libraries, while cDNA DGED uses the publicly available UniGene Expression and Gene relational databases to count the sequences found for each gene in the presented libraries.

**Results:**

We discovered that the CGAP approach often includes libraries from dependent or irrelevant tissues (one third of libraries were incorrect on average, with some tissue searches no correct libraries being selected at all). We also discovered that the CGAP approach reported genes from outside the selected libraries and may omit genes found within the libraries. Other errors include the incorrect estimation of the significance values and inaccurate settings for the library size cut-off values. We advocated a revised approach to finding libraries associated with tissues. In doing so, libraries from dependent or irrelevant tissues do not get included in the final library pool. We also revised the method for determining the presence or absence of a gene by searching the UniGene relational database, revised calculation of statistical significance and sorted the library cut-off filter.

**Conclusion:**

Our results justify re-evaluation of all previously reported results where NCBI CGAP expression data and tools were used.

## Background

Gene expression profiling is a powerful approach for identifying alternatively expressed genes, as in the case of tumour markers or for studying tissue, organ or cellular specificity or time- and course-dependent gene expression profiles. Traditional methods suitable for studying mRNA expression include Northern blots [1], DNA arrays [2] and quantitative PCR [3]. Expressed sequence tag (EST) expression profiling is another well-established method used to acquire quantitative information on a sample's transcriptome. ESTs are produced by randomly sequencing clones in a cDNA library, usually from the 3' end to generate single read fragments which are then assembled into longer, overlapping sequences mapped onto the original transcript. The current version of the UniGene database [4] contains 123,459 individual entries for Homo sapiens (2,520,273 total entries, last accessed 14 January 2011). Serial analysis of gene expression (SAGE) is another high throughput method for the analysis of gene expression patterns, differing from EST in that only short sequence tags are sequenced to uniquely identify a transcript and no other sequence information is collected [5]. Huge amount of experimental data requires new approaches to storing, annotating and accessing the data. Examples of relevant databases include ArrayExpress Archive [6], Gene Expression Omnibus - a public functional genomics data repository [7] and Cancer Genome Anatomy Project (CGAP) - cancer specific information and a suite of informatics tools [8]. Because of the high redundancy of EST and SAGE data, these could be used to assess the abundance of relevant transcripts and gene expression changes in response to stimuli or under pathological conditions. The number of ESTs sequenced from a sample that align and map onto each transcript can be counted to produce a representative profile of gene expression in the sample from which the cDNA library was created. Differential gene expression between two samples can therefore be detected from variations in the tag counts for a specific transcript, normalised relative to the size of each sample [9]. Large-scale gene expression data constitute a valuable resource but the sheer scale of the datasets requires dedicated informatics tools and may be challenging.

Run by the US National Cancer Institute (NCI) since 1996, the CGAP project aims to generate the information and technological tools needed to decipher the molecular anatomy of the cancer cell [10]. Since then CGAP tools were used widely in the analysis or for validation of the differential gene expression in e.g. brain cancers and retinoblastomas [11-14], breast cancer [15-17], colon cancer [18-24], gastric cancer [25], lung cancer [26], pancreatic cancer [24,27,28], prostate cancer [29,30] and haematological malignancies [31] to name just a few. The improved methods of analysing and mining this data include NCBI classification system based on hierarchically related keywords, assigned to each new library by NCI staff. Furthermore, CGAP hosts two bioinformatics tools, the cDNA xProfiler [32] and the cDNA Digital Gene Expression Displayer (DGED) [33], which are designed to enable a user to identify differentially expressed genes, e.g. between a cancer and a normal tissue, or compare gene expression between two user-selectable pools of libraries.

**Figure 1:**
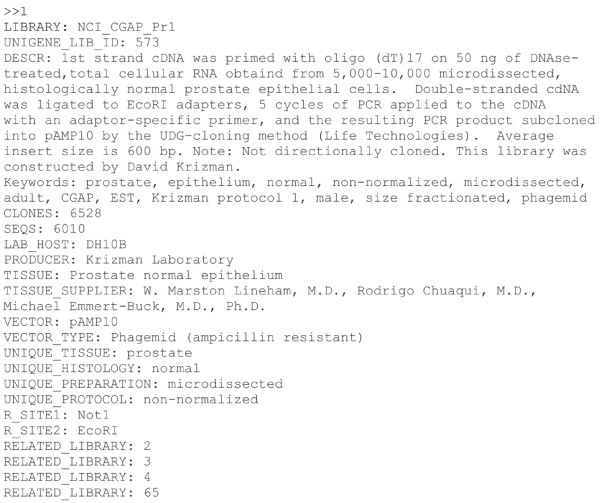
**CGAP library database entry**. Each field entry begins with its heading, shown in capital letters, followed by the value associated with that field, after the colon.

Both tools search the UniGene repository [34], a publicly available relational EST library database maintained by the US National Center for Biotechnology Information (NCBI) in which the tag counts from submitted human or house mouse EST libraries are mapped onto UniGene IDs, the unique transcripts they most closely match. NCBI uses tissue type/sample type annotation to create an ontology hierarchy in which libraries are grouped into tissue types according to tissue dependency (bone marrow, for example is a constituent of bone tissue, so bone marrow libraries are listed under bone tissue). The user-submitted tissue type annotations are listed for each library under the "keywords" and "unique tissue" fields, see Figure 1, which shows the first entry of the UniGene EST library database.

The CGAP tools use the values associated with the "keywords" field, to include or exclude libraries from a search based on the chosen tissue type and the inclusion of any dependent tissues under the selected one in CGAP's ontology hierarchy. Using this field, which also includes information on a library's histology, libraries from a secondary tumour (for example, neuroblastoma which has metastasised to bone marrow) can also be listed under the tissue in which the primary tumour is located (brain in the case of neuroblastoma) [35,36].

cDNA DGED relies on the UniGene relational database maintained by NCBI, whilst the cDNA xProfiler accesses a flat file database, not available for on-line access (personal communication from Carl Schaefer, National Cancer Institute, Center for Biomedical Informatics and Information Technology, USA), and uses a Boolean type search to identify the presence or absence of a gene in either or both of two groups (pools) of libraries which the user has chosen to compare to find differentially expressed genes. It lists the results as a table detailing how many matching genes have a known or unknown name and/or function (listed as known or unknown) and how many are found only in the libraries in the two pools or in at least one library outside the two pools (listed as unique or non-unique [37], also reviewed in [35].

Although the presence of a transcript in a particular library can be revealing, the outcome would depend on many parameters, including the size of the libraries used, and is therefore of limited biological significance. cDNA DGED uses a similar on-line user interface to allow seamless selection of the two pools of libraries, calculates sequence odds ratio for individual genes expressed in the two pools [38] and calculates the statistically significance for each result. cDNA DGED yields the most biologically relevant prediction - the normalised odds ratio, which at least in principle should be comparable to the results obtained through other methods based on Northern hybridisations or DNA microarrays. The user interface is straightforward and the simple calculation principle appears reassuringly reliable. Underlying data are also available in raw data format allowing the use of alternative tools for the data interrogation.

In the course of our work we tried to replicate xProfiler and cDNA DGED algorithms in our quest to further improve them. To our surprise we found that not only xProfiler yields different gene lists compared to cDNA DGED when all the same parameters are used, but also that the core hierarchical classification system on which both xProfiler and DGED are based isn't flawless. We have therefore decided to have a closer look at the database query and data analysis approached available on the CGAP server. To this end we identified errors in gene lists generation, library classification by tissue, calculation of statistics, and library database records. We have also reported our findings to NCBI in the hope that these problems will be corrected quickly for the benefit of the scientific community.

## Results

### Errors in CGAP tools

#### Errors in library search algorithm used by CGAP tools

**Figure 2:**
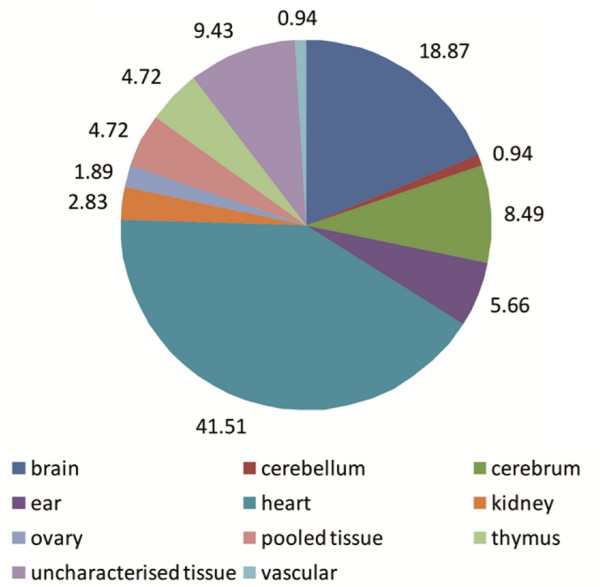
**Tissue type origin of libraries reported for by CGAP tools after searching for "ear" tissue**. All the libraries reported in this search (database access date 13 May 2010) were then manually checked for their "unique tissue" annotations and the percentage of the reported libraries which originate from all tissues were calculated.

In our attempt to replicate xProfiler and cDNA DGED algorithms we found that the core hierarchical classification system on which both xProfiler and DGED rely isn't flawless. For example a search of cDNA database for the "ear" tissue resulted in over 100 libraries of which only six were actually generated from ear or related tissues (see Figure 2, database access date 13 May 2010). The remaining ~94% of the libraries would be from irrelevant tissues such as the heart and brain. Other tissues also contained irrelevant libraries, e.g. brain library pool contained nine other unrelated tissues, or if eye libraries were selected, out of the 73 libraries, five were mixed tissues. Table 1 reports the correct/incorrect library inclusion rates for all other tissue types available and listed on the CGAP server. We believe that the CGAP library selection algorithms had serious flaws; we detail these below and in Figure 2, using the "ear" library search as an example.

**Table 1:** Error rates for the CGAP library selection tools

Tissue types available	Percentage of correctly reported libraries	Percentage of incorrectly reported libraries
Adipose	100.00	0.00
Adrenal cortex	100.00	0.00
Adrenal medulla	100.00	0.00
Bone	33.04	66.96
Bone marrow	96.43	3.57
Brain	53.29	46.71
Breast/Mammary Gland	99.39	0.61
Cartilage	100.00	0.00
Cerebellum	92.86	7.14
Cerebrum	99.53	0.47
Cervix	100.00	0.00
Colon	98.88	1.12
Ear	5.66	94.34
Embryonic tissue	8.45	91.55
Endocrine	5.62	94.38
Eye	61.11	38.89
Gastrointestinal tract	3.47	96.53
Genitourinary system^a^	0.00	0.00
Germ cell	11.54	88.46
Head and neck	0.42	99.58
Heart	51.76	48.24
Kidney	94.31	5.69
Limb	0.00	100.00
Liver	83.66	16.34
Lung	97.27	2.73
Lymph node	100.00	0.00
Lymphoreticular	14.16	85.84
Mammary gland/Breast	99.39	0.61
Muscle	25.71	74.29
Nervous	0.92	99.08
Oesophagus	95.45	4.55
Ovary	95.92	4.08
Pancreas	67.35	32.65
Pancreatic islet	100.00	0.00
Parathyroid	57.14	42.86
Peripheral nervous system	12.50	87.50
Pineal gland	87.50	12.50
Pituitary gland	93.33	6.67
Placenta	99.48	0.52
Pooled tissue^b^	Not available	Not available
Prostate	97.46	2.54
Retina	100.00	0.00
Salivary gland	62.50	37.50
Skin	89.00	11.00
Soft tissue	1.74	98.26
Spleen	78.57	21.43
Stem cell	33.72	66.28
Stomach	94.07	5.93
Synovium	100.00	0.00
Testis	98.67	1.33
Thymus	97.50	2.50
Thyroid	97.57	2.43
Uncharacterised tissue	99.75	0.25
Uterus	99.22	0.78
Vascular	91.89	8.11
White Blood Cells^b^	0.00	0.00

^a ^No libraries were present in the database for these tissues

^b ^Pooled tissue was not available in the CGAP tools, which listed these libraries under each of the tissues they were produced from.

All libraries containing a text string "heart" in their "keywords" field seem to be included indicating that CGAP search for the correct string "ear" using any text matches, regardless of whether that string is part of a longer string such as "heart" or is a standalone word (as in ear tissue). This deficiency also brings into the results some libraries whose "unique tissue" field contains "brain", "cerebellum", "cerebrum", "thymus" or "vascular" because their "keywords" contain the phrase "heart disease" in their "keywords" field. This results in the inclusion of dependent or irrelevant tissues.

Other heart libraries which do not contain "heart" in their "keywords" field but contain "pericardium" instead are still included despite the fact that the letters "ear" do not appear in their "keywords" field. We found these to contain "heart" in their "unique tissue" field. Therefore CGAP must be searching "unique tissue" field similarly to the "keywords" fields and erroneously include partial text string matches.

A number of other libraries that contain "kidney" or "ovary" in their "unique tissue" field were included. We identified the reason for these - their "keywords" field contains text "clear cell renal carcinoma" or "clear cell ovarian tumor", where a search string "ear" can be found in "clear".

Another keyword field error for "ear" search was found for libraries whose "unique tissue" field contains "uncharacterised tissue". Under their "keywords" field we found text "peripheral blood mononuclear cell" which is an incorrectly match for the search string "ear".

Finally, and unexpectedly, libraries created from mixed tissue samples (and therefore contain "pooled tissue" in their "unique tissue" field) were still included even if they did not contain the ear tissues. The reason is the same as described above - these libraries contained "heart" in their "keywords" field.

Yet another error type is related to cancer metastasis. Two particular examples are the inclusion by CGAP under brain of a bone library which contains the phrase "Ewing's sarcoma" in its "keywords" field and the inclusion of five bone marrow libraries which contain "neuroblastoma" in their "keywords" field. The inclusion of the Ewing's sarcoma library is erroneous because Ewing's sarcoma is known to be a bone condition. However, this keyword could be searched for because, as was recently discovered, in extremely rare cases Ewing's sarcoma will metastasise to the right front parietal scalp, which is adjacent to the frontal and parietal lobes of the right cerebral hemisphere. The inclusion of the bone marrow libraries is erroneous because these are made from secondary metastases of the primary neuroblastoma, which is located in brain tissue [36,39].

#### Errors in CGAP's gene search algorithm

**Figure 3:**
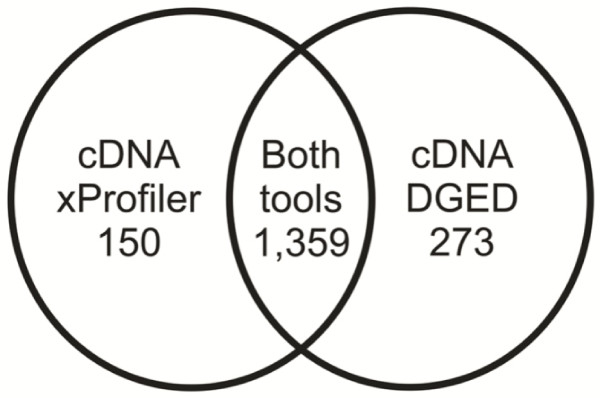
**Differences in the number of genes reported by CGAP tools for an identical query**. The total number of genes reported to be present when normal adipose libraries (in one pool) are compared with cancerous bone libraries (in the other pool) by xProfiler's gene lists (left circle) and cDNA DGED (right circle). The overlap between the two circles represents the genes reported by both tools.

We also found that xProfiler yields different gene lists compared to cDNA DGED when all the same parameters are used. For example when a normal adipose tissue was compared with cancerous adipose tissue using xProfiler and cDNA DGED, 1,359 genes were reported by both tools, 150 additional genes were reported by xProfiler only and 273 by cDNA DGED only, see Figure 3. This problem was not limited to this tissue alone. We also discovered that the xProfiler reports additional genes to be present in its summary table of gene results, compared to the gene lists, see Table 2. As this table also shows, the total number of genes reported by cDNA DGED is greater than the number reported by the xProfiler's gene lists and less than the number reported by the xProfiler's results table. We tried to analyse these discrepancies by looking into gene annotations for the genes which were listed incorrectly, i.e. not listed by all the tools.

**Table 2:** Number of genes reported to be present in both pools when normal adipose libraries (in one pool) were compared with cancerous adipose tissues (in the other pool), by xProfiler's gene lists and summary table of gene results, and by cDNA DGED.

Tool and output method used	Number of genes reported
cDNA xProfiler results table	1,688
cDNA xProfiler gene lists	1,509
cDNA DGED	1,632

The correct list of genes to report for this particular comparison of normal adipose tissue with cancerous adipose tissue should include both the 1,359 genes reported by both tools and the 273 genes reported only by cDNA DGED. Both our gene search routine and CGAP DGED appear to produce correct gene lists whilst xProfiler missed 273 genes and also incorrectly selected 150 genes. This is because cDNA DGED accesses the UniGene relational database using the same method as our algorithm (see "Gene search algorithm" in the methods section) to find genes which are represented by sequences in one or more of the libraries presented in each pool, whilst xProfiler accesses a flat file database which appears to miss 273 genes from the presented libraries and also incorrectly lists as being present in the chosen libraries the 150 additional genes. This discovery was confirmed by closer inspection of the results for the comparison of normal and cancerous bone libraries shown in Table 3, which revealed that xProfiler reported 237 additional genes not reported by cDNA DGED, while 707 of the genes reported by cDNA DGED were omitted from xProfiler's results.

**Table 3:** Number of genes reported to be present in either or both pools when normal bone libraries (in one pool) are compared with cancerous bone libraries (in the other pool) by xProfiler's gene lists and summary table of gene results, cDNA DGED and using our algorithm.

Tool and output method used	Number of genes reported
*Reporting the presence or absence of each gene in a Boolean manner*	
cDNA xProfiler results table	10,108
Our algorithm	9,996

*Reporting the sequence odds ratio for each gene*	
cDNA DGED	9,996
Our algorithm	9,996

#### Problem with CGAP statistics

The probability values "P" reported by the DGED are based on Bayesian statistics [38], and are detailed below(1)

where P is the probability value reported by cDNA DGED and L is calculated as follows:(2)

where F is the sequence odds ratio, which is calculated as follows:(3)

where "a" is the number of sequences representing the gene concerned from all libraries in Pool A, "A" is the number of sequences representing all genes across all libraries in Pool A; "b" is the number of sequences representing the gene in question in all libraries included in Pool B and "B" is the number of sequences representing all genes in all libraries in Pool B.

The value "P" should indicate the reliability of the calculated odds ratio "F". We found that "P" values calculated by CGAP DGED would change depending on the user-selected display cut-off for the odds ratio "F". This is certainly incorrect, as the probability of finding upregulation should not depend on whether a whole list of genes or part of that list is looked at. We believe that probability of the result being correct should not depend on the display cut-off setting. Table 4 illustrates this for three distinct entries (all three are upregulated more than threefold). If the display cut-off value "F" is set to two, all three "P" values are reported as zero or very close to zero (in the manner of a Chi-squared "P" value [40]), which indicates statistically very significant results. However, if the display cut-off value "F" is set to three, the "P" values for all three results increase, indicating apparently reduced statistical significance, which is not the case.

**Table 4:** Change in probability values "P" reported by cDNA DGED and our algorithm when the display cut-off value "F" is changed are exemplified for three genes that are presented in the gene list when normal bone libraries are compared with cancerous bone libraries

UniGene Cluster ID	Name	Symbol	**CGAP "P" values**^**a**^		**Our "P" values**^**b**^	
			
			"F" = 2	"F" = 3	"F" = 2	"F" = 3
164226	Thrombospondin 1, mRNA	THBS1	0.001	0.049	0.978	0.978
369397	CDNA FLJ53400 complete cds	TGFBI	0.001	0.045	0.982	0.982
462998	In-IGFBP-4 mRNA	IGFBP4	0.000	0.007	0.999	0.999

^a ^Calculated using on-line tools from CGAP; calculations based on equations (1 - 3) in this report). The calculated "P" value is close to zero (on a scale of zero to one) if the probability is high that the observed expression difference is genuinely greater than the user-specified "F" value, and is not due to sampling error [31,33].

^b ^Calculated using equation (4) in this report. This produces a "P" value of between zero and one, but unlike the CGAP value, this is a decimal fraction of the likelihood of there being at least a threefold difference in expression of the transcript in the activated cells.

#### CGAP incorrectly calculates number of sequences per library

The number of sequences in each library is an indirect indicator of the library quality because a library containing only a few sequences is less likely to provide a representative picture of gene expression in the sample from which it was created than a library in which many sequences map onto genes. As Table 5 shows, when we compared normal adipose tissue with cancerous adipose tissue using cDNA DGED, we discovered that the sum of the number of sequences in each library based on the annotations in the library database (in the "sequences" field, see Figure 1) was always greater than the number of sequences cDNA DGED reported to be mapped onto all the genes in each pool. This problem is not limited to adipose tissue and it affects the majority of the library database.

**Table 5:** Total number of sequences reported for normal adipose tissue libraries and for cancerous adipose tissue by cDNA DGED library list and gene list

Number of sequences reported	Sequences in normal adipose libraries	Sequences in cancerous adipose libraries
Library list	2,285	1,740
Gene list	1,799	721

### Our solutions

In the attempt to identify the causes of errors and to further improve xProfiler and cDNA DGED algorithms we looked into and revised library and gene parsing algorithms.

#### Solution to the errors in the library search algorithm

We have designed a library parsing algorithm to search only for the exact tissue type in each library's "unique tissue" field. For example, if ear is selected, we select libraries which only contain the exact string "ear" in this field and which do not have any other annotations in this field, resulting in the selection of libraries from the chosen tissue type without the inclusion of libraries from dependent or irrelevant tissues.

If the required phrase is part of a longer phrase in a library's annotation (for example the phrase "bone" is part of the "bone marrow" annotation in the "unique" tissue field of a bone marrow library), the library with the longer phrase is ignored and not included in the results. In this example the selection would only contain bone libraries and not include bone marrow libraries. Our algorithm does this by searching for the required phrase as the only annotation in the "unique tissue" field, which does not contain any information other than the correct tissue type annotation for each library and therefore does not select dependent or irrelevant tissues.

#### Solution to the errors in the gene search algorithm

We also devised two gene parsing algorithms which search the UniGene relational database (as does cDNA DGED) for genes contained within the presented libraries. One reports the expression information for each gene as a Boolean type result identifying the presence or absence of a gene in a pool (as does xProfiler), while the other calculates a sequence odds ratio for each individual gene. Both report the results as a single list of all the genes present in at least one pool along with the expression information. As Table 3 shows, these algorithms report the same gene counts. cDNA DGED reports the same gene count for the same set of libraries whilst xProfiler does not.

#### Solution to the problem with CGAP statistics

Though we could not completely implement cDNA DGED's statistics, we solved the CGAP problem of "P" values changing after changing the display value "F" by implementing the method described by Chen et al [41] and used by Boon et al [42]. That method calculates the probability of threefold upregulation in one pool compared to the other. However we have left the "P" values on a scale of zero to one and have not converted them to a percentage scale.(4)

where P is the probability value reported by our novel algorithm, A is the number of sequences reported for a gene in Pool A and B is the number of sequences reported for a gene in Pool B. Table 4 shows three genes from a comparison of normal bone tissue libraries with cancerous bone tissue libraries; the values do not change when the "F" value display cut-off is changed.

#### Solution to the problem with number of sequences per library

We solved the problem of sequences being reported by CGAP tools for each library which did not map onto the UniGene transcripts within that library. We calculated the number of sequences in each library which map onto the genes reported for that library, and add this information to the library database for reporting in the list of libraries. Our library parsing algorithm uses this calculated number instead of the "sequences" figure submitted by the library creator and included in the database by CGAP. As Table 6 shows, our approach reports the same total for the number of libraries in each pool as it does for the number of sequences which map onto the transcripts in that pool.

**Table 6:** Total number of sequences reported for normal bone libraries and for cancerous bone libraries by the library list and gene list produced by CGAP tools and by our routine.

Number of sequences reported	Sequences reported for normal bone libraries	Sequences reported for cancerous bone libraries
Library list from CGAP tools	19,308	18,197
Gene list from cDNA DGED	17,844	16,635
Library list from our new algorithm	17,844	16,635
Gene list from our new algorithm	17,844	16,635

## Discussion

In our quest to understand and improve the xProfiler and cDNA DGED algorithms we chose Microsoft Excel to re-create xProfiler and cDNA DGED algorithms. Although this software is not designed for regular gene expression analysis, we found it suitable for or the construction of a tool to show proof of principle for our algorithms. As explained in this report, we have used this program to rectify the errors in the CGAP algorithms and to prove that we can produce the correct gene expression data from the correct set of libraries for each tissue and for identifying true gene expression levels.

### Errors in library search algorithm

The CGAP library parsing algorithm appears to search for libraries which contain the required tissue name in their "keywords" field regardless of whether that tissue name (a text string) is part of a longer phrase (part of a longer text string) such as "clear cell ovarian tumor" and regardless of whether their "unique tissue" field states a relevant or irrelevant tissue origin. This results in massively inaccurate choice of libraries and could easily lead to the selection of completely irrelevant libraries and yield artificial differences in gene expression and false disease markers. This is a major problem, which went undetected for many years and which require re-evaluation of all previously reported results where NCBI CGAP expression data and tools were used. CGAP creators allowed for the additional manual control of the choice of libraries before the gene expression data are obtained. But even this feature might not be practical for larger library collections, such as e.g. brain (over 1,000 libraries), or e.g. "uncharacterised tissue" (over 2,000 libraries, of which over half actually contain detailed descriptions with sufficient data for library classification).

As Table 1 shows, the CGAP hierarchical classification system also appears to consider libraries made from secondary tumours which have formed by metastasis of the primary tumour in the tissue in question, as belonging to that tissue. When brain tissue is selected, libraries are included from nine irrelevant tissues, including bone and bone marrow. The bone library in question was created from a Ewing's sarcoma sample. Its inclusion under brain tissue is therefore erroneous, although it has been recently discovered that Ewing's sarcoma will metastasise to the right front parietal scalp [39], which is adjacent to the frontal and parietal lobes of the right cerebral hemisphere. Generally speaking, when a secondary tumour forms it will present a significantly different gene expression profile from the primary tumour due to the different gene expression profile of the secondary tumour's location. Hence, for the purpose of gene expression analysis, secondary tumours should not be considered as belonging to the tissues they metastasised from.

The suggested amendments implemented in our algorithm solve the problems by searching the contents of each library's "unique tissue" field, with the result that our tool groups only the correct libraries to the chosen tissue type. The effect of this is that any genes found to be differentially expressed between normal and cancerous libraries from that tissue will be genuine tumour markers because they are differentially expressed only in the specified tissue, and are not false positive results that are due to the impact of libraries from other tissues on the expression data, as could be the case with the CGAP results.

Once our findings were reported to NCBI this error in CGAP's library parsing algorithm was corrected. Currently both xProfiler and DGED algorithms (last accessed on 10 January 2011) search for libraries which contain the phrase for the chosen tissue in their "unique tissue" field and ignore libraries which contain this string as part of a longer string within this field. However, the CGAP parsing tools would still erroneously include libraries created from mixed tissue samples if their "keywords" annotations contain the required text phrase for the chosen tissue.

### Errors in CGAP's gene search algorithm

We have also investigated the reasons as to why the list of xProfiler genes differ from the list obtained by DGED, as illustrated for adipose tissue in Figure 3. The internally available flat file database accessed by the cDNA xProfiler was found to show differences in the genes present in each library when compared with the publicly available UniGene relational database. We could not find an explanation as to why this is so, given that not all genes which are reported by only one tool are related to those which are reported by both tools as shown in Table 7. The analysis of gene annotations revealed that xProfiler incorrectly lists cDNAs which are absent from the library pool, but which have names or functions similar to the genes present in the designed library pool. The effect of this is that, even if the list of libraries for the chosen tissue is correct (as they are for tissues such as adipose, as Table 1 shows), the gene list could still include false positive differentially expressed genes or omit valid tumour markers which could otherwise warrant further investigation for use in cancer diagnosis or as a novel target for anticancer therapy.

**Table 7:** Data from UniGene relational database for α-actinin genes reported by CGAP xProfiler and/or cDNA DGED tools for a comparison of a pool containing normal adipose libraries with a pool containing cancerous adipose libraries.

Tool that reported gene in either or both pools	Gene Symbol	Gene Title	UniGene Cluster ID
cDNA DGED only	ACTN4	Actinin, alpha 4	270291
cDNA DGED and cDNA xProfiler	ACTN1	Actinin, alpha 1	509765

Our gene parsing algorithms solve the problems associated with CGAP's xProfiler algorithm by reporting only the UniGene transcripts which sequences in each library map on to, thus reporting the same genes regardless of whether the output format is Boolean or includes the sequence odds ratios, as Table 3 shows. Now that the library parsing algorithm has also been corrected, this will ensure that the reported genes do not include false positive differentially expressed genes or omit genuine tumour markers which could otherwise be investigated further. Since we reported our findings to NCBI this error has been corrected. Both tools (last accessed 10 January 2011) show identical numbers of genes when all the same parameters are used.

### Problem with CGAP statistics

The statistics used by cDNA DGED and which are calculated using Equation (1), which was based on the earlier reported approach and Equation (4). Although the latter does not depend on the display cut-off value for "F", the former includes "F" and therefore makes the calculated probability "P" that a transcript is unregulated in one pool compared to the other, dependent on the display setting. We believe this is an error, contributing to the false discovery rate of tumour markers or the omission of potentially valid markers.

Although we could not reproduce exactly the Bayesian statistics implemented by cDNA DGED [38], we have implemented the original, previously reported statistical method on which the CGAP statistics were supposedly based. Our approach is based on Equation (4) and it allowed us to calculate the probability that the level of the expression of a given transcript is increased by at least threefold in one pool compared to the other. The output is given on a scale of zero to one, such that a transcript with a 95% probability of threefold upregulation in one pool compared to the other is given a "P" value of 0.95. These values do not depend on the "F" ratio display cut-off setting. As Table 4 shows, our method yields the same "P" values regardless of the chosen display cut-off values.

The original Bayesian statistics calculations on the CGAP website have now been replaced with two tests. cDNA DGED calculates "P" values for each gene using the Fisher Exact Test. These are then converted to "Q" values using the Benjamini Hochberg False Discovery Rate. These "Q" values are reported. However when last checked on 12 January 2011 the reported "Q" values would still change depending on the user-selected display cut-off for the odds ratio "F".

### Problem with number of sequences reported

We have also looked into why the number of sequences as annotated in the library database is greater than the number of sequences which map onto the genes in the library, as illustrated for adipose tissue in Table 5. This difference in the "sequences" annotation when compared to the "number of sequences representing genes in library" annotation could not explain the differences in the gene lists produced by the CGAP tools and was thought to arise from the fact that some of the sequences in the library did not map onto genes when the library was originally sequenced.

Also, although the user can filter the libraries by size (by setting the minimum number of sequences per library), the CGAP tools use the sequences annotation in the library database (see Figure 1) to implement such a cut-off, rather than the number of sequences which map onto the transcripts in the library. The CGAP approach produces results which are less reliable than they initially appear because, although the sequences annotation in the library database may be greater than the chosen cut-off value, the number of sequences mapping onto the transcripts in the library may actually be below the cut-off.

We have calculated the actual number of sequences which map onto a library's transcript and programmed our library parsing algorithm to apply the sequences display cut-off to this value rather than the sequences annotation of each library, which includes sequence which do not map onto genes. As Table 6 shows our algorithm reports the same total number of sequences in the library list as it does for the gene list if the chosen output format shows the sequence odds ratios, which in turn is the same as the value reported by CGAP's cDNA DGED for the same libraries. The effect of this is that the user can more accurately apply this to determine the reliability of the reported libraries, for a library in which few sequences map onto genes is less likely to provide a representative profile of gene expression in the sample from which it was created than a library in which many sequences map onto genes. Furthermore, the display cut-off will not take into account any sequences which do not map onto genes, so it can be used reliably to determine the quality of the results.

NCBI have not yet implemented a solution to this problem in the CGAP library and gene parsing algorithms (last checked on 12 January 2011). The sum of the number of sequences per library annotations (in the "sequences" field, as reported by CGAP's library parsing algorithm) is still greater than the number of sequences the gene parsing algorithm of cDNA DGED reports to be mapped onto all the genes in each pool at the top of the gene expression table.

## Conclusions

In conclusion, we were genuinely surprised to learn that apparently simple process of reporting differential gene expression patterns between two tissue samples depended so much on the algorithms used to select the libraries and genes for the analysis and on the calculations of the "P" values. We found that the selection of libraries for different tissues is dependent on the libraries' annotations and the parsing techniques which have to interpret these often inaccurate annotations. We also found that the results were equally dependent on the selection of genes and on the validity of the reported number of sequences in the libraries.

• Our library parsing algorithm groups libraries to the chosen tissue type by searching the contents of each library's "unique tissue" field, instead of the "keywords" field. The algorithm searches only for the selected tissue and does not search for dependent or irrelevant tissues.

• Our gene parsing algorithm searches the relational UniGene database for genes included in the chosen libraries, regardless of whether the user chooses to report the results in Boolean format or with the sequence odds ratios included.

• Our assessment of the reliability of differential gene expression is based on the method reported by Chen et al [41].

• Instead of using the "sequences" value supplied by CGAP for each library to implement the display cut-off for the number of sequences in each library, one should count the number of actual sequences which map onto each transcript in the library

The probability values "P" reported by the DGED, have been replaced by newly devised false discovery rate "Q" parameter, which, is not dissimilar to the "P" and is the important reminder that the expression odds ratio must be interpreted with caution, but nothing warns the user of the sometimes more severe problems of cDNA libraries' annotations and parsing. Following our discoveries, we have alerted CGAP creators of the discrepancies, and some of these problems have been sorted. The problem with CGAP's statistical calculations still remains (even though the statistics have recently been updated) as does the problem with the reporting of the number of sequences per library.

When last checked on 10 January 2011 CGAP's library parsing algorithm is still imperfect and requires further attention. For example, if either xProfiler or cDNA DGED are used to compare normal adipose tissue with cancerous adipose tissue, no libraries are reported despite the fact that adipose libraries will be presented if all normal tissues are compared with cancerous adipose using the same settings.

Our results justify critical re-evaluation of the current database retrieval and expression analysis tools at NCBI CGAP. These expression databases and tools were available since 1996 and all of the bioinformatics and/or cancer research, where NCBI CGAP expression data and tools were used, may need to be revisited.

## Methods

### The use of CGAP tools and CGAP search settings

#### Library searches

For each of the 56 available tissues in CGAP's database a search was carried out in which all criteria were set to present as many libraries as possible (using the version of CGAP's library parsing algorithm available on 14 May 2010). Tissue type was set to the tissue under investigation. All the libraries presented for each tissue were manually checked to assign each library as correctly or incorrectly reported. A library was considered to be correctly reported if its "unique tissue" annotation precisely matched the selected tissue type. Any libraries whose "unique tissue" annotations did not precisely match the selected tissue type were considered to be incorrectly reported. No libraries contained any of the phrases "germ cell," "head and neck" or "stem cell" in their "unique tissue" field, so libraries were considered to be correctly reported for these tissues if they contained these phrases in their "keywords" field. All libraries that mapped onto each tissue type were reported, regardless of whether they contained a representative profile of *in vivo *gene expression.

#### Gene lists

To check the gene lists produced by the CGAP tools normal adipose tissue and cancerous adipose tissues were compared using both xProfiler and cDNA DGED (using the version of these tools available on 16 March 2010). Bulk and non-normalised libraries were used. The number of sequences display cut-off was set to zero to include all libraries. When running searches using cDNA DGED the Bayesian probability "P" value and the calculated odds "F" ratio display cut-offs were both set to one to ensure that all genes were displayed to enable comparison of the results with those produced by xProfiler, which does not have statistical filters.

#### Gene searches for revealing problem with CGAP statistics

To test two different "F" value display cut-off settings, two CGAP's cDNA DGED searches were run to compare normal bone with cancerous bone (accessed on 9 January 2010). As with the adipose search described above, bone libraries were chosen from bulk tissue samples and were libraries which had not been normalised during their preparation. The number of sequences display cut-off was set to zero to include all libraries. The "P" value display cut-off was set to one for both searches to present all results regardless of their reliability. The "F" value display cut-off was set to two for the first search to display all genes whose expression differed between the two pools of libraries by a factor of two or more. The "F" display cut-off was set to three for the second search to display every gene whose expression differed between the two pools by a factor of three or more. The output of the on-line search results was analysed for three genes whose "P" values were at or close to zero when the "F" value cut-off was set to two and compared these "P" values with those obtained when the "F" value display cut off was set to three.

#### Gene searches conducted to reveal problems with number of sequences reported

The comparison of normal adipose tissue with cancerous adipose tissue described above was repeated using CGAP's cDNA DGED (the database was last accessed on 6 January 2010). The number of sequences contained within the libraries of each pool was counted for each pool (by summing together the values reported for the individual libraries from CGAP's library database annotations). This value was compared with the value reported by CGAP DGED gene list for the number of sequences representing all genes in the chosen libraries.

### Replication and fail proofing of the search algorithms

#### Library search algorithm

We used Microsoft Office Excel 2007 to test our library parsing algorithm. Initially this was designed to mimic CGAP tools and present the same libraries for each tissue as do the CGAP tools, so we could compare our gene parsing algorithm (see" Gene search algorithm") with that offered by CGAP. We were therefore able to compare our "number of sequences representing gene in library" values with the "sequences" values reported by CGAP. Without this we would not be able to report any differences in the gene results compared to the CGAP tools as being solely due to differences in the gene parsing algorithm between the tools. We then modified our algorithm to assign libraries to tissues using their "unique tissue" field to present only the libraries which are associated with the selected tissue. All libraries for each tissue were reported, regardless of whether they contained a representative profile of *in vivo *gene expression. The library searches described above were repeated for each available tissue using our algorithm (the relevant UniGene databases were downloaded from CGAP website on 2 January 2010), whilst keeping all the other settings the same.

#### Gene search algorithm

Also using Microsoft Excel, two new gene search routines were designed. One reports the presence or absence of that gene in each pool of libraries, as reported by xProfiler. The other reports the number of sequences representing that gene in all of the libraries included in each pool and calculates the sequence odds ratio for each gene between the two pools, as does cDNA DGED. Both algorithms rely on the UniGene Library ID (the unique identifier) of each of the chosen libraries in the expression datasheet of the UniGene relational database used by cDNA DGED. This table lists the genes in each library along with the number of sequences in that library which represent each of those genes. The UniGene Cluster ID (the unique identifier) of each gene, which is used to identify it in the expression datasheet, is used to search the gene datasheet for the details of that gene. These are reported in a gene list. The presence or absence of each of the presented genes in each pool of libraries and the number of sequences representing that gene in all pooled libraries are reported.

We tested our gene parsing routines by comparing a set of normal bone libraries with a set of libraries from cancerous bone. The chosen libraries were made from bulk bone tissue and had not been normalised during their preparation, thus matching as closely as possible the *in vivo *gene expression levels. The libraries used were the same as those presented by the on-line CGAP tools for bone tissue, in order to show that any differences in the gene results were due to differences in the gene parsing algorithms and not due to differences in the library parsing algorithms. The number of sequences cut-off was set to zero to include all qualifying libraries of any size.

#### Solution to the problem with CGAP statistics

Statistical methods implemented in CGAP DGED for calculating a Bayesian probability value for each gene [38] are based on the statistical methods reported earlier [41]. We could not reproduce exactly the statistics reported by Lal et al [37] so the original statistics reported by Chen et al. [41] and used by Boon et al [42] was implemented instead (see Equation (4)). We used that equation to calculate the probability of the threefold upregulation of a transcript when a group of normal bone libraries was compared with a group of cancerous libraries (the relevant UniGene databases were downloaded from CGAP website on 2 January 2010). We used the same libraries as those the existing CGAP tools map onto bone tissue. The definite integral shown in Equation (4) was calculated using 32-point Gaussian quadrature, a method for efficient and highly accurate evaluation of definite integrals [43]. The "P" values calculated using Equation (4) range between zero and one. The statistically significant values are those closest to one (for example, a result might be significant and the null hypothesis rejected if the "P" value is 0.95 or greater). In [41] this value is converted to a percentage, with statistically significant values being those closest to 100%, but they are presented in our novel algorithm on a scale of zero to one, as Table 4 shows. To check whether the "P" values calculated using Equation (4) change when the display cut-off value "F" is changed, the bone searches mentioned earlier were repeated and the gene parsing algorithm was run twice; once with the "F" value display cut-off set to two and once with the "F" value display cut-off set to three.

#### Solving the problem with number of sequences reported per library

We routinely counted for each library the number of sequences representing all the genes in that library, and compared these values to the ones reported by both CGAP tools' library lists and the number of sequences representing the genes in the same libraries as reported by cDNA DGED's gene list. In particular, data presented in Table 6 were obtained by comparing our calculated "number of sequences representing genes in library" values for a pool of normal libraries from bone tissue and a pool of cancerous libraries from bone tissue (using the UniGene database downloaded from NCBI on 15 November 2008) with the "sequences annotations" for the same libraries reported by CGAP tools (using the version of xProfiler's available on 1 December 2008 and the version of cDNA DGED available on 27 November 2008. The chosen libraries were produced from bulk tissue samples and had not been normalised during their processing, to ensure that the results provided a representative profile of *in vivo *gene expression. The sequences display cut-off was set to zero to ensure that the new values could be compared with the CGAP values.

## Authors' contributions

ATM carried out most of the experimental work and contributed to the drafting of the manuscript. MS conceived and supervised the study, and drafted the manuscript. Both authors read and approved the final manuscript.

## Response

By Kenneth Buetow and Carl Schaefer

E-Mail: buetowk@nih.gov

Address: Laboratory of Population Genetics, National Cancer Institute, National Institutes of Health, Bethesda, MD 20892

The authors report an error in the CGAP web site: searches for libraries by tissue type could, in some cases, return erroneous results. In June of 2006 the search code was incorrectly changed from exact string matching to substring matching. Since the substring match did not respect word boundaries, there were in some rare instances wrong inferences where substrings are nested within larger words. For example, "ear" would match "heart". While not as obvious, this is also part of the underlying explanation for the anomalous retrieval of "bone marrow" and "Ewing's sarcoma" in response to a query on "brain". In these latter cases, the query "brain" matched the intermediate-level term "brain/CNS tumor" in the histology portion of the keyword hierarchy provided by NCBI. Since the terms "neuroblastoma" and "Ewing's sarcoma" are descendants of "brain/CNS tumor" in the NCBI hierarchy, they were selected as appropriate matches for the (inappropriately) substring-matched "brain/CNS tumor". It would be usual to classify Ewing's sarcoma and neuroblastoma as peripheral tumors rather than central, and it is precisely because the logic of ontologies such as the NCBI keyword hierarchy is sometimes obscure, if not simply wrong, that the CGAP cDNA XProfiler and cDNA DGED present the keywords of retrieved libraries to the user and allow the user to explicitly include or exclude a given library.

A second error noted by the authors, different gene inventories identified by similar searches in the cDNA XProfiler and cDNA DGED, resulted from the query having been issued during a window between the updating of the database tables on which the cDNA DGED relies and the updating of a cached flat file on which the cDNA XProfiler relies.

A third issue, failure to retrieve results for "adipose", was brought to our attention only when we read the authors' submission to prepare this response. This failure is due to an error in the HTML of the user interface. The value for the selection "adipose" in the pulldown menu should be "adipose tissue" rather than simply "adipose".

We are grateful to the authors for discovering and reporting these problems, all of which were corrected within a day of their being reported. With respect to other matters discussed in the paper, we do not agree with the authors' conclusions.

While the CGAP project has been in existence since 1996, the CGAP web site and associated web queries were developed, hosted and maintained by NCBI prior to 2001.

The CGAP web site imports flat files produced by NCBI. These files are filtered, joined, and reformatted in various ways; some of the resulting processed data is imported into the CGAP relational database and some of the resulting processed data is retained in flat files. However, CGAP does not, as stated by the authors, query NCBI's UniGene relational database directly.

The library search employed by the cDNA Xprofiler and the cDNA DGED does not search on the unique tissue field. The unique tissue designation for each library is not imported directly from NCBI. Rather it is computed by combining three sources of information: the keywords supplied explicitly provided by NCBI, the keyword hierarchy provided by NCBI, and the tissue selectors in the CGAP web site user interface. It is this combination that allows the CGAP tools to conclude that a library with the keyword "pericardium" should be retrieved in response to a query on "heart" selected from a predefined menu of tissues. For some tools on the CGAP website, including the cDNA XProfiler and the cDNA DGED, it was decided to allow a direct search on keywords rather than restricting the search to the unique tissue designation. And for this very reason, those tools interpose a library selection page between the initial tissue query and the computation of results, as noted by the authors. This library selection page, which provides the user a complete list of libraries, with keywords, in each of the two library pools, allows the user to remove libraries from the query or to change a library from one pool to the other.

The authors contend that the cDNA DGED and the cDNA XProfiler should report only the total number of sequences in a library that map to UniGene clusters rather than the total number of sequences generated from a library. Presumably, the authors would use the smaller total in computing the relative abundance of a gene. We do not consider the suggested approach to be more accurate. On the contrary, if a library has 10,000 sequences and if half of these map to UniGene clusters, and if one UniGene cluster is represented by 100 sequences, it seems to us more accurate to report an abundance of 1/100 rather than 1/50 for that cluster.

In the Bayesian computation of significance used previously by the cDNA DGED, the function of the F parameter was not, as the authors state, to serve as a display filter. Rather, it served as the null hypothesis against which significance was computed. If F = 2, then the null hypothesis is that a given gene is twice as abundant in one pool as in the other pool. Clearly, if the null hypothesis (F) is changed, then the resulting p-value will change. In principle, the Bayesian computation was better suited to the analysis task than the Fisher Exact, which had been used for several years before being replaced by the Bayesian computation. However, in a number of cases, the implementation of the Bayesian computation failed to converge. This is the reason that the CGAP web site reverted to the Fisher Exact. As a measure of significance, the cDNA DGED now reports a q-value. The Benjamini-Hochberg q-value of an observation is a function of the rank position of the p-value of the observation in an ordered sequence of p-values for the observations under consideration. If an observation does not meet the initial odds ratio (or fold change) threshold, then a p-value is not computed. So changing the odds ratio threshold will change the set of observations under consideration, which will change the ordered set of p-values, which can change the q-value of a given observation.

As noted at the start of this reply, the anomalous difference in gene inventories between the cDNA XProfiler and the cDNA DGED was due to a stale cached flat file. It had nothing to do with "gene parsing algorithms". As the authors are undoubtedly aware, the inventory and constitution of UniGene clusters can change dramatically from one build to the next.

We would like to call attention to a simple and inexpensive method of reporting bugs (real or apparent) with the CGAP web site: this is the "Application Support" link that appears at the bottom of every page on the CGAP site and that has been available to user for more than ten years.

In summary, we find the conclusion drawn by the authors that all historic analysis performed using the CGAP site should be in question, is not supported by the data.

Kenneth H. Buetow, Ph.D.

Carl F. Schaefer, Ph.D.
